# Having a Say Matters: Influence of Decision-Making Power on Contraceptive Use among Nigerian Women Ages 35–49 Years

**DOI:** 10.1371/journal.pone.0098702

**Published:** 2014-06-04

**Authors:** Funmilola M. OlaOlorun, Michelle J. Hindin

**Affiliations:** 1 Department of Preventive Medicine & Primary Care, College of Medicine, University of Ibadan, Ibadan, Nigeria; 2 Department of Population, Family & Reproductive Health, Johns Hopkins Bloomberg School of Public Health, Baltimore, Maryland, United States of America; University Hospital of Münster, Germany

## Abstract

**Background:**

Research suggests that women of reproductive age who are involved in household decision-making are more likely than those who are not involved to be able to control their fertility. Little is known, however, about this relationship among women at the upper end of the reproductive spectrum. The aim of this study was to determine the association between household decision-making power and modern contraceptive use among Nigerian women ages 35–49 years.

**Methods:**

A descriptive, cross-sectional study involving a secondary analysis of data from the Nigerian 2008 Demographic and Health Survey was conducted among women ages 35–49 years who were considered to be in need of contraception. The outcome was modern contraceptive use while the main independent variable was a woman's household decision-making power score, constructed using principal component analysis. Multivariate logistic regression was performed to determine whether the women's household decision-making power score, categorized into tertiles, was independently associated with modern contraceptive use. Data were weighted and adjusted for the complex survey design.

**Results:**

Prevalence of modern contraceptive use among Nigerian women deemed to be in need of contraception in this study was 18.7%. Multivariate logistic regression showed that women's decision-making power remained statistically significantly associated with modern contraceptive use, even after adjusting for age, education, religion, polygyny, parity, wealth and domicile. Women who were in the highest decision-making power tertile had more than one and a half times the odds of using modern contraception compared with women in the lowest tertile [Adjusted Odds Ratio = 1.70; 95% Confidence Interval = 1.31–2.21, p<0.001].

**Significance:**

Older Nigerian women who are involved in making household decisions are also able to make decisions related to their fertility. Programs in Nigeria focused on increasing modern contraceptive use should include strategies to increase women's status through encouraging more visible involvement in decision-making across different spheres of their lives.

## Introduction

Women ages 35 and older are often left out of the conversation on contraception for a variety of reasons including their waning fecundity, their age or perceived infertility, or the belief that they have sexual intercourse infrequently, and so are not at risk of pregnancy. Researchers have known for several decades that even though many of these women wish to avoid pregnancy, they are unlikely to be using any contraceptive method, or, if using, opt for unreliable methods. [1] Some older women erroneously believe they are not at risk of pregnancy, and therefore do not need modern contraception. [1], [2] Women, as they approach menopause, often experience irregular periods for many years before their periods finally cease, and so do not have the monthly reminder of their fecundity. It is important that these older women are protected against unplanned pregnancies, especially since they have a greater risk of pregnancy-related morbidity and mortality when compared with their younger counterparts. [3]

There are a wide variety of factors that have been associated with contraceptive-related choices and decisions. One of these factors is a woman's decision-making power within the household. There is a body of literature that suggests that women who are actively involved in domestic decision-making are able to control their fertility through the adoption of modern contraception. [4]–[7] Modernization of many subcultures within Nigeria, like in other developing settings [8] allows women to receive higher education; actively engage in the labor force; marry at older ages; have their first birth at older ages; choose their own partners; and live apart from extended families. This has helped to reduce the control of relatives over couples and their decisions, and has been associated with greater female decision-making power in some settings. [9], [10] While several researchers have examined the role of decision-making power in contraceptive use, this has been done across the full reproductive age spectrum. [11], [12] Most studies look at all women of reproductive age and do not provide effect estimates for older women separately, often including age as a continuous variable in their multivariable models, or grouping all women 35 years and older into a single category, despite the heterogeneity this introduces.

There is an impressive body of literature on contraceptive use, or the lack thereof in resource-constrained settings. Less attention has been paid, however, to women at the upper end of the reproductive age spectrum who have been described as a special [13] and overlooked [14] population. Women ages 35–49 represent 12.7% of the female population and 6.2% of the total Nigerian population. [15] Nigerian women ages 35 and older who are still menstruating are of special interest because they tend to be left out of existing family planning programs and services which currently target teenagers, women who attend antenatal and postnatal clinic, and men. Although data are available on these women through the Demographic and Health Survey (DHS) report, no published study to date has addressed the role older women's involvement in decision-making may play in contraceptive use.

### Fertility, contraceptive use, and household decision-making within the Nigerian context

Nigeria is a large and very diverse country with a population of over 160 million and about 389 different ethnic groups. Over 70% of the Nigerian population resides in rural areas, yet resources (trained personnel, infrastructure, public social amenities, allocation of funds, etc.) are largely concentrated in the urban areas. The total fertility rate is 5.7 children per woman and slightly higher than the total wanted fertility rate of 5.3 children per woman. Total fertility rates are higher in rural (6.3) than urban areas (4.7) and in the north (North Central -5.4; North East - 7.2; North West -7.3) than the south (South West -4.5; South South - 4.7; South East - 4.8). [16] Current modern contraceptive prevalence rate is 10.5% among Nigerian women 15–49 years, with the method mix consisting largely of the male condom, injectables and oral pills. [16] Current use of modern contraception exhibits a large north-south divide with prevalence being much higher in the south (South West-21.0%; South South-15.5%; South East-11.8%) than in the north (North Central-10.5%; North East- 3.5%; North West-2.5%). Unmet need remains high at 20% across all age groups of women.

Nigeria remains a patriarchal society where men continue to dominate all spheres of women's lives, a cultural norm fiercely protected within traditional institutions. [17] The National Gender Policy notes that the rights of Nigerian women are undermined and undervalued, [17] and this often serves as an impediment to women who aspire to pursue their careers and attain both management and decision-making positions at the same pace as their male colleagues. Today in Nigeria, women, especially those who are educated and older, are slowly rising to important decision-making roles at the local, state and national levels. This wave of change may in time influence women's decision-making within their households, and in all spheres of their lives, including the ability to negotiate reproductive preferences that will suit their changing lifestyles. One such lifestyle change is the desire to limit family size using modern contraception. Little is known, however, about how involvement in household decision-making is associated with use of modern contraception among older Nigerian women. This information will not only help us to better understand older women's contraceptive experiences, as well as social and contextual factors that may influence their decisions or restrict their choice, but will also provide a basis to put forward policy and programmatic recommendations that will ensure the contraceptive needs of this unique group of women are met.

This study will begin to address this gap in knowledge by: (1) Describing the profile of women ages 35 and older who are current modern contraceptive users; (2) Comparing household decision-making power among modern contraceptive users and non-users; and (3) Examining the association between women's decision-making power and current modern contraceptive use among Nigerian women ages 35–49 years. We hypothesized that women's decision-making power would be independently associated with current modern contraceptive use, even after adjusting for potential confounding factors.

## Methods

### Data

This paper uses data from the 2008 Nigerian National Demographic and Health Survey (NDHS), a nationally representative survey of 33,385 women aged 15–49 and 15,486 men aged 15–59. (Data available at: http://dhsprogram.com/data/dataset/Nigeria_Standard-DHS_2008.cfm?flag=0) The sample for the survey was selected from all six geo-political zones, including a proportional sample of both urban and rural dwellers of each of the 36 states and the Federal Capital Territory in the six geopolitical zones from June to October, 2008. Using a stratified two-stage cluster design, 888 clusters (286 urban, 602 rural) were selected, providing a representative sample of 36,800 households. In the second stage, an average of 41 households was selected per cluster, using equal probability systematic sampling. All women aged 15–49 years in selected households who were permanent residents or visitors present in the households on the night before the survey were eligible to be interviewed. [16]

The survey included among other topics detailed information about fertility, nuptuality, sexual activity, fertility preferences, awareness and use of family planning methods. Data were collected using three questionnaires – the Household Questionnaire, the Women's Questionnaire, and the Men's Questionnaire. The instruments were pre-tested in six states and the questionnaires were amended based on the pre-test findings. Questionnaires were translated into the major local languages (Hausa, Igbo, Yoruba), and back-translated into English to ensure the original meaning was retained.

### Analytic sample

Women interviewed in the NDHS who were 35–49 years of age and currently married or living with a man were eligible to be included in the analysis, irrespective of current contraceptive use status. Women were excluded because they were not currently married or living with a man (998; 10.2%); wanted another child within 24 months (2,122; 21.6%); were pregnant (588; 6.0%); or had not seen their menses for 6 months (or since last birth if this was within a year of the survey date), provided they were not using contraception. Of the 9831 women aged 35 and older, 4827 were deemed to be in need of contraception and thus eligible for the present analyses.

### Variables

The outcome of interest was current use of any modern contraceptive method (sterilization, pill, intrauterine device, injectable, implant, condom, diaphragm, foam/jelly, emergency contraceptive), which was recoded as a dichotomous variable (currently using coded “1”, not currently using a modern method/currently using a traditional/folkloric method coded “0”).

Women's decision-making power was the key independent variable and assessed based on women's responses to six separate questions: (1) “Who usually decides how the money you earn will be used?” (2) “Who usually decides how your husband's/partner's earnings will be used?” (3) “Who usually makes decisions about health care for yourself?” (4) “Who usually makes decisions about making major household purchases?” (5) “Who usually makes decisions about making purchases for daily household needs?” (6) “Who usually makes decisions about visits to your family or relatives?” For each of these questions, a woman was given five options: (1) respondent (2) husband/partner (3) respondent and husband/partner jointly (4) someone else (5) other. These variables were further re-categorized and coded in accordance with the research questions. For decisions regarding a woman's earnings or that of her husband/partner, the coding was as follows: “0” respondent/partner had no earnings “1” respondent/partner had earnings but respondent had no say in how they were spent “2” respondent had a say in how her earnings or that of her husband/partner were spent. The other four variables were binary and coded “0” if the respondent had no say in the decision and “1” if she had a say.

Having a say in a decision implied that the woman decided alone or jointly with her husband/partner. A household decision-making power score was calculated based on a woman's responses to all of these questions using principal component analysis (PCA) which lends itself very well to data reduction in situations such as this (see Joliffe and Morgan, 1992). [18] Since women's decision-making power is multi-dimensional in nature, [19] PCA was employed to replace these six variables with a single new variable, while minimizing loss of information. Based on the number of principal components with eigenvalues greater than 1, a screeplot, and parallel analysis, the data yielded a single factor or principal component. Tertiles were constructed from the score variable to provide a measure of relative household decision-making power.

Other independent variables of interest in this analysis included those that were conceptually recognized to be potential confounders of the association between decision-making power and contraceptive use among this sub-population of women. (A) Individual factors: (1) Age was analyzed in 3 year age groups (35–37y; 38–40y; 41–43y; 44–46y; 47–49y). (2) The total number of children ever born was categorized as “0–2 children”; “3–4 children”; “5–6 children”; and “7+ children”. (3) Polygyny was analyzed as a binary variable (polygynous union or not). (4) Educational level was categorized as “no formal”; “primary”; “secondary” and “tertiary”. (5) Religion was coded as “Catholic”; “Other Christian”; “Muslim”; “Others”. (B) Household factors: (1) Household wealth - The wealth index, a measure of relative economic well-being based on household assets was categorized into quintiles (lowest, second, middle, fourth, highest) and derived from the wealth score. (2) Domicile - Place of residence was dichotomized into “urban” or “rural” since the profile of women and availability of services are known to differ in these two settings.

### Ethics statement

The study was deemed to qualify for ethical review exemption by the Johns Hopkins Institutional Review Board.

### Data analyses

Data were explored and frequencies and cross-tabulations generated to understand the patterns of distribution of the variables of interest. Simple and multivariate logistic regression models were constructed to examine the unadjusted and adjusted effects of women's decision-making power on current modern contraceptive use among Nigerian women ages 35–49 years. The final model tested whether women's decision-making power was independently associated with current modern contraceptive use among this subset of women. Post-estimation statistics showed that the model was a good fit for the data. To check for multicollinearity, variance inflation factors were generated and were less than 10 for all variables in the final model. Multivariate logistic regression analysis used weighted maximum likelihood estimation with an adjusted Wald test F statistic. The estimated standard errors of the log odds ratios were adjusted for the complex survey design by the Taylor linearization method.

Sensitivity analyses were done to assess the robustness of the findings. Specifically, women who reported being sterilized and women less than 40 years of age were removed from the analytic sample, and the regression models were re-run. Analysis were run without sterilized women to confirm that these women who had opted for a permanent form of contraception, and were therefore at no risk of unintended pregnancy, did not disproportionately drive the observed association between decision-making and contraceptive use. We excluded women who were less than 40 years because we wanted to be sure that our choice of 35 years as a cut-point was justified. We felt women under 40 years may have less motivation to avoid pregnancy than their counterparts over 40. All analyses were conducted using STATA version 12 (College Station, TX, USA).

## Results

The prevalence of use of any family planning method among women in our sample was 27.4% (modern-18.7%; traditional-7.1%; folkloric-1.6%). Of the women who were currently using a modern method, the method mix included injectables (6.0%); intrauterine device (IUD; 3.3%); oral pills (3.0%); male condom (2.9%); female sterilization (1.6%); norplant (0.1%); and others (1.8%). Traditional methods used were periodic abstinence (4.2%) and withdrawal (2.9%). Contraceptive use increased with education and declined with age. Report of modern contraceptive use was most frequent among other Christians, women with 3-4 children and those not in polygynous unions. Regarding household level variables, modern contraceptive use increased with wealth and was higher in urban than rural areas. The full socio-demographic profile of respondents can be found in Table 1.

**Table 1:** Socio-demographic Profile of Nigerian Women by Current Modern Contraceptive Use Status.

		Users	Non-users	
Variables		%	%	p value
**Total**	**n**	**839**	**3988**	
**Age group (years)**				
35–37 y	1,280	30.4	25.7	0.001
38–40 y	1,426	30.9	29.3	
41–43 y	643	13.7	13.2	
44–46 y	891	16.1	19.0	
47–49 y	587	8.9	12.8	
**Educational level**				
No formal education	2,248	17.3	52.7	<0.001
Primary	1,247	32.2	24.5	
Secondary	938	33.0	16.6	
Higher	394	17.5	6.2	
**Religion**				
Catholic	487	10.5	10.0	<0.001
Other Christian	1,930	64.0	34.9	
Islam	2,284	23.7	52.3	
Traditionalist	126	1.8	2.8	
**Polygamous union**				
No	2,917	75.1	57.4	<0.001
Yes	1,910	24.9	42.7	
**Children ever born**				
0–2 children	208	2.0	4.8	<0.001
3–4 children	849	26.2	15.8	
5–6 children	1,367	38.3	26.2	
7+ children	2,403	33.5	53.2	
**Wealth tertile**				
Poorest	1,022	6.0	22.9	<0.001
Poorer	986	9.7	22.2	
Middle	936	18.8	20.2	
Richer	915	26.1	18.7	
Richest	968	39.5	15.9	
**Domicile**				
Urban	1556	48.9	28.7	<0.001
Rural	3271	51.1	71.3	

Women who were current modern contraceptive users were more likely to be involved in household decision-making in all six domains examined, when compared with their counterparts who were not currently using modern contraception (Table 2).

**Table 2:** Women's Involvement in Household Decision-making by Current Modern Contraceptive Use Status.

Items	Users	Non-users	P value
	% (95% CI)	% (95% CI)	
**Her earnings** †			
No income	9.4 (7.3, 11.5)	19.2 (17.6, 20.8)	<0.001
Not involved in decision	20.7 (17.4, 24.0)	23.8 (21.9, 25.7)	
Involved	69.9 (66.3, 73.6)	57.0 (54.7, 59.3)	
**His earnings**			
No/unknown income	2.0 (1.0, 3.0)	2.0 (1.5, 2.5)	<0.001
Not involved in decision	52.2 (48.2, 56.1)	67.9 (65.8, 70.0)	
Involved	45.8 (41.9, 49.8)	30.1 (28.1, 32.2)	
**Her healthcare**			
Not involved in decision	27.6 (24.3, 30.9)	50.6 (48.5, 52.8)	<0.001
Involved	72.4 (69.1, 75.7)	49.4 (47.2, 51.6)	
**Major purchases**			
Not involved in decision	37.1 (33.7, 40.6)	57.5 (55.4, 59.6)	<0.001
Involved	62.9 (59.4, 66.3)	42.5 (40.4, 44.6)	
**Daily purchases**			
Not involved in decision	20.9 (17.9, 23.8)	43.4 (41.2, 45.6)	<0.001
Involved	79.1 (76.2, 82.1)	56.6 (54.4, 58.8)	
**Visiting friends & family**			
Not involved in decision	20.1 (17.2, 22.9)	39.2 (36.8, 41.6)	<0.001
Involved	79.9 (77.1, 82.8)	60.8 (58.4, 63.2)	
**Statistics for decision-making power scale**			
n	839	3988	
Mean (SE) of scale	0.80 (0.06)	−0.17 (0.03)	
T statistic; p value	−14.09; p<0.001		

† Note: Chi square for trend: 63.61; p<0.001.

Multivariate logistic regression showed that women's decision-making power remained statistically significantly associated with modern contraceptive use, even after adjusting for potential confounders (see Table 3). Women who had the most decision-making power had more than one and a half times the odds of using modern contraception compared with women in the lowest tertile of decision-making [Adjusted Odds Ratio (aOR) (95% Confidence Interval) (95% CI): 1.70 (1.31, 2.21), p<0.001]. Older women (ages 41–43; 44–46; 47–49) had lower odds of reporting current use of modern contraceptives when compared with women ages 35–37 years [aOR (95% CI): 0.67 (0.50, 0.90); 0.73 (0.55, 0.98); 0.59 (0.43, 0.82) respectively]. Similarly, women in polygynous unions had lower odds of reporting current use of modern contraceptives when compared with women in monogamous unions [aOR (95% CI): 0.80 (0.65, 0.98)]. Other Christians (non-Catholics) had higher odds of modern contraceptive use compared with their Catholic counterparts [aOR (95% CI): 1.84 (1.30, 2.60)]. Muslims and Traditionalists were no different from Catholics in terms of modern contraceptive use. Wealthier women had higher odds of modern contraceptive use compared with their poorer counterparts [aORs (95% CI) for the middle, richer and richest women were respectively 2.06 (1.41, 3.01); 2.50 (1.67, 3.76); 3.47 (2.22, 5.44)]. Compared with women who had 0–2 children, women with more children had higher odds of modern contraceptive use [aOR (95% CI) for women with 3–4, 5–6 and 7 or more children were respectively 2.35 (1.35, 4.07); 2.81 (1.64, 4.84); 2.22 (1.25, 3.93)].

**Table 3:** Odds Ratios and 95%CI of Current Modern Contraceptive Use among Nigerian Women (35–49y).

	Unadjusted OR (95% CI)	Adjusted OR (95% CI)
**Decision-making power**		
Lowest tertile	1.00	1.00
Middle tertile	2.48 (1.94, 3.16)	1.33 (1.02, 1.73)
Highest tertile	3.70 (2.93, 4.67)	1.70 (1.31, 2.21)
**Age group (years)**		
35–37y	1.00	1.00
38–40y	0.92 (0.74, 1.15)	0.95 (0.75, 1.20)
41–43y	0.78 (0.59, 1.02)	0.67 (0.50, 0.90)
44–46y	0.70 (0.53, 0.90)	0.73 (0.55, 0.98)
47–49y	0.58 (0.43, 0.79)	0.59 (0.43, 0.82)
**Educational level**		
No formal education	1.00	1.00
Primary	3.33 (2.56, 4.34)	1.85 (1.38, 2.47)
Secondary	4.88 (3.67, 6.48)	1.86 (1.30, 2.64)
Higher	6.58 (4.74, 9.13)	2.37 (1.59, 3.54)
**Religion**		
Catholic	1.00	1.00
Other Christian	1.85 (1.35, 2.53)	1.84 (1.30, 2.60)
Islam	0.61 (0.43, 0.87)	1.05 (0.70, 1.57)
Traditionalist	0.91 (0.46, 1.78)	1.64 (0.80, 3.38)
**Polygamous union**		
No	1.00	1.00
Yes	0.49 (0.40, 0.59)	0.80 (0.65, 0.98)
**Children ever born**		
0–2 children	1.00	1.00
3–4 children	2.61 (1.52, 4.49)	2.35 (1.35, 4.07)
5–6 children	2.44 (1.43, 4.15)	2.81 (1.64, 4.84)
7+ children	1.19 (0.68, 2.07)	2.22 (1.25, 3.93)
**Wealth quintile**		
Poorest	1.00	1.00
Poorer	1.36 (0.93, 1.99)	1.14 (0.79, 1.66)
Middle	2.84 (1.95, 4.14)	2.06 (1.41, 3.01)
Richer	4.23 (2.89, 6.18)	2.50 (1.67, 3.76)
Richest	7.19 (5.02, 10.28)	3.47 (2.22, 5.44)
**Domicile**		
Urban	1.00	1.00
Rural	0.46 (0.38, 0.55)	0.87 (0.69, 1.11)

In the sensitivity analysis which excluded women less than 40 years, and for which women aged 40 were the reference group with age analyzed in single years, all older women had lower odds of reporting current modern contraceptive use when compared with women in the reference group. The relation between age and contraceptive use was only statistically significant for women ages 47–49 years (results not shown), with women 41–46 years being no different from 40-year old women, holding constant other variables in the model. In both scenarios, the results followed the same pattern as the full sample, there were no differences in the direction of estimates, and only minimal changes in the effect sizes.

## Discussion

We assessed the relation of household decision-making and current modern contraceptive use among Nigerian women ages 35–49 years. In accordance with our hypothesis, women's decision-making power was independently associated with current modern contraceptive use, even after adjusting for potential confounding factors. While other researchers have also found that women's decision-making power is an important determinant of modern contraceptive use, [5]–[7], [20] this study adds to the existing body of research by providing a focus on women ages 35–49. Unlike many other studies, this study focuses on an understudied group of women 35 years and older, who are expected to have acquired the ability to negotiate their reproductive desires with their partners over time and are believed to have greater motivations for contraceptive use than their younger counterparts.

Modern contraceptive use among women ages 35–49 years in this analytic sample (18.7%) appears to be higher than that for women 15–49 years in the study sample (10.5%), but it is important to recognize that these two figures are not directly comparable since the former sample only includes women deemed to be in need of contraception while the latter sample includes all women, irrespective of their need for contraception. The finding that contraceptive use is significantly lower among women older than 40 years when compared with women 35–40 years is probably related to the perception that with reduced sexual exposure and reduced fecundity, the risk for pregnancy is low, making contraceptive use unnecessary. These women, however, based on their self-reported fertility desires continue to need contraception to avoid unwanted pregnancies, as long as they remain sexually active. Qualitative studies will be useful to further explore motivations for use and non-use among older Nigerian women.

Findings from this study reveal that having a say in household decision-making matters for older Nigerian women with regard to their use of modern contraception. The results also suggest that a Nigerian woman's involvement in household decision-making is important, no matter what the decision is. This suggests that our choice of statistical technique to represent the multidimensional nature of decision-making power was justified. Women's decision-making power has been conceptualized and measured by different researchers in many different ways, and there is still no consensus on how best to define and measure this construct. In addition, comparisons between studies are difficult because of the different ways researchers have defined similar, yet different terms such as “autonomy”; “empowerment”; “agency”; status”; “power”; etc. (See Malhotra et al., 2002 for a detailed discussion of the diversity and similarities in the existing literature on women's empowerment.) [21] A limitation of this study is that the data are restricted to decision-making power, so it is possible that other dimensions of empowerment may have different associations with modern contraceptive use.

While the cross-sectional nature of this study makes it difficult to assert the temporal ordering of events, it is only logical to think that women who were involved in household decision-making were very different from those who were not. Such “decision-makers” may have developed a sense of self-agency over time, and learned to assert themselves within their households. In addition, these older women belong to a generation whose mothers were unlikely to have had access to modern contraception, and who probably did not know about modern contraception in their teenage years. Thus, the decision to use modern contraception may be seen in these older women as a bold step or a deviation from what they were taught to be the norm regarding childbearing.

In this analysis, the number of children ever born, educational attainment and household wealth accounted for some of the association observed between decision-making and modern contraceptive use. These are characteristics that give women social stature in the Nigerian context where, for instance, children are highly valued, all women are expected to have children of their own, and infertile women are assumed to be infertile due to previous induced abortions. [22] The fear of infertility following modern contraceptive use has been reported as a barrier to uptake. [23] Education and wealth command the respect of others and have been widely reported to be associated with both women's household decision-making [24], [25] and their use of modern contraception. [12], [26] This suggests that women who were of high social standing also reported greater involvement in household decision-making and were able to control their fertility through the use of modern contraception.

This study has focused specifically on women ages 35–49 years, and the findings are only generalizable to women of this age in Nigeria and similar settings who are deemed to be in need of contraception. Although the choice of the age “35” is arbitrary, it is in recognition and appreciation of the difference between social age, which is concerned with the different roles a person takes along their lifecycle [27], [28] and chronological age, the number of years lived. The socially constructed nature of age tends to vary across context and time in history. [28] While some women at a chronological age of 35 have what may seem like an older social age because they started childbearing early, others want more children at this age and are yet to attain their desired family size. Women ages 35 and older often think they do not need contraception even though they are sexually active, have a regular sexual partner, have not attained menopause and do not desire a pregnancy. However, all women, irrespective of age, who are sexually active, do not desire a pregnancy, are not sterilized and have not attained menopause need contraception.

Women's decision-making power within their households enables them to make more personal decisions regarding their reproductive health, such as the use of modern contraception. This suggests that when women are encouraged to be involved in the decision-making process with their partners, they become empowered to make other decisions about their reproductive health, including the use of modern contraceptives. Programmatic efforts and policies to increase modern contraceptive use among women should be cognizant of the different life stages women pass through. Programs should include interventions to improve a woman's ability to negotiate with her partner regarding involvement in decision-making within the household, including decisions related to her fertility. Policies should be formulated in such a way as to recognize that women 35 and older are a unique group with contraceptive needs that may differ from their younger counterparts due to a different life stage.

Gender equality needs to become well engrained in the Nigerian culture, so that men will always treat their partners' decision-making opinion as important. Health care providers and opinion leaders such as priests, clergy men and imams (Muslim clerics) need to disseminate clear, culturally appropriate messages that will help women and their partners understand that older women may still be fertile and need to use contraception if they remain sexually active and are not prepared for the risks associated with pregnancy in older women.
